# Effect of the Type of Lateritic Soil on the Effectiveness of Geomechanical Improvement Using a Low Quantity of Cement for Sustainable Road Construction

**DOI:** 10.3390/ma16216891

**Published:** 2023-10-27

**Authors:** Marie Thérèse Marame Mbengue, Abdou Lawane Gana, Adamah Messan, Ousseni Mone, Anne Pantet

**Affiliations:** 1 Laboratoire Eco-Matériaux et Habitats Durables (LEMHaD), Institut International d’Ingénierie de l’Eau et de l’Environnement (2iE), 1, Rue de la Science, Ouagadougou 01 BP 594, Burkina Faso; therese.mbengue@2ie-edu.org (M.T.M.M.); abdou.lawane@2ie-edu.org (A.L.G.); 2 Laboratoire Ondes et Milieux Complexes (LOMC), Université Le Havre Normandie, UMR 6294 CNRS, 76063 Le Havre, France; anne.pantet@univ-lehavre.fr; 3 ACIT Géotechnique, Ouagadougou 12 BP 217, Burkina Faso; moneousseni@yahoo.fr

**Keywords:** lateritic soils, geomechanical properties, cement addition, physical properties, base layer, road construction

## Abstract

In Burkina Faso, the most commonly used road construction material is lateritic soil. However, in its raw state, this soil does not meet the required recommendations. To overcome this problem, previous studies have often focused on improving these soils by adding cement. However, these studies have rarely included a multi-criteria characterisation of the main geomechanical parameters of treated soils. It was also noted that the identification parameters of lateritic soils could have an influence on their improvement with cement. The aim of this study is to highlight the influence of the physical and mineralogical properties of lateritic soils on the effectiveness of improving their geomechanical properties by adding a low content of cement (<3% wt.). The soils were taken from two sites: Saaba (LAS) and Kamboinsé (LAK). The effects of cement addition on the plasticity index, CBR index, Young’s modulus, unconfined compressive strength, tensile strength and shear strength were studied. In their raw state, LAS and LAK have different physical properties and cannot be used as sub-bases. The addition of cement improves the overall physical and mechanical properties of both soils, but to different degrees. Indeed, after adding 3% cement to the raw soils, the CBR index of LAS increases by 1275% compared with 257% for LAK; the unconfined compressive strength of LAS is twice as high as that of LAK, and the Young’s modulus increases by around 460% for LAS compared with 360% for LAK. After improvement, these two soils met all the CEBTP specifications except for tensile strength. The effect of cement was more significant on LAS due to its better physical properties and higher clay mineral content, which would improve pozzolanic reactivity during cement hydration. Knowing the mineralogy of lateritic soils when treating them with cement would allow us to reduce the quantity of cement, thereby mitigating its negative impact on the environment.

## 1. Introduction

The construction of road structures requires the availability of sufficient quantity and quality of materials for pavements. In subtropical Africa, there are high-performance materials that can be used in road construction, such as aggregates derived from magmatic rock. However, these materials are rarely used, as they require expensive means of extraction and transport. The lateritic soils, which are particularly prevalent, remain the material of choice for road construction. In Burkina Faso, these soils cover almost two-thirds of the country [1], are relatively inexpensive to exploit because they are easy to extract and are often located close to the infrastructure site. These materials are derived from the intense weathering of rocks in tropical conditions and are used for the construction of subgrade, foundations, base layers and even embankments. Their geotechnical properties are not variable because they are influenced by climate, geology and the degree of weathering [2,3], as well as their varying degrees of friability. As a result, the road infrastructures built with these materials do not achieve their projected lifespan, given their premature deterioration [4]. The conditions of use, with excessive traffic loads, partly explain this deterioration. The use of lateritic soils is conditioned by traditional specifications [5,6,7,8]. Most of these specifications are based on considerations from the identification tests, but rarely on the mechanical parameters of the material.

Most studies on lateritic soils have focused on their physical characterisation, their pedogenetic classification, sometimes on their mineralogical composition and rarely on their mechanical characterisation (compressive strength, elastic modulus, and shear parameters that condition the development of permanent deformations responsible for ruts on pavements). For road structures that have to carry heavy traffic, most lateritic soils in their natural state are only suitable as sub-bases [9]. Two main techniques are used to improve the performance of these soils for use as sub-base or base layers: mechanical stabilisation and chemical stabilisation. Mechanical stabilisation involves improving the granular skeleton of a soil by adding aggregates in order to increase the compactability and mechanical strength of the soil [10,11,12,13,14,15,16,17,18,19]. Chemical stabilisation involves modifying the geotechnical properties of the soil through a chemical reaction between additives (generally, lime and/or cement) and the fine particles, especially clay minerals in the soil. This method is very widespread in subtropical Africa [20,21,22,23,24,25,26].

The mechanism for stabilising soils with cement involves a series of chemical reactions between clay minerals, cement and water. There are two main reactions: cement hydration and secondary pozzolanic reactions. Cement hydration leads to the formation of primary cement-based products. Hydrated calcium silicate compounds (C-S-H) bind the components of the clay-cement mixture, thus making a significant contribution to improving the mechanical properties of the mixture. In addition, portlandite (Ca(OH)_2_) is produced. During the second reaction, the portlandite, water, silica and alumina react to produce various cementing compounds. Silica and alumina can be derived from clay minerals, quartz, feldspars, micas, alumino-silicate minerals, and even certain amorphous compounds. The presence of a significant amount of lime in soil significantly increases the pH of the mixture to 12.4, which corresponds to the pH of a solution saturated with lime. The high pH causes the silica in the clay minerals to dissolve. This dissolved silica combines with the Ca^2+^ ions released by the portlandite to produce hydrated calcium silicate and/or hydrated calcium aluminate [23,24]. It was shown that this technique can improve the mechanical performance of these soils by changing their initial low bearing capacity class to a higher bearing capacity class. It allows the use of raw soil that was only suitable as a backfill layer as a base layer for pavements after treatment. However, the properties used to assess the improved properties of these soils are often based on bearing capacity (CBR), tensile strength and compressive strength [22,23,24,25,27]. Moreover, the studies carried out on these soils only examine the influence of the addition of high cement contents (more than 3% of cement by weight) on their physical and mechanical properties. The use of high cement contents leads to semi-rigid pavements [25,28], which may result in early cracking of pavements in sub-tropical countries. In addition, the use of high cement contents leads to environmental pollution. Cement is not only an imported material in some countries, but its production is also responsible for 5 to 7% of the world’s carbon dioxide (CO_2_) emissions, and CO_2_ accounts for 65% of greenhouse gases. Reducing the use of cement should therefore be strongly encouraged for low-traffic pavements in order to mitigate global change. However, the effect of adding a low content of cement (less than 3% of cement by weight) to different types of laterites has rarely been studied. According to [25], the improvement of lateritic soils by the addition of cement (less than 3% cement) compared to stabilisation (greater than 3% cement) can present an economical and effective solution for the design of flexible pavements.

This study aims to assess the effect of adding a low content of cement on the physical (grain size distribution and plasticity limits) and mechanical properties (CBR index, unconfined compressive strength, elasticity modulus direct and direct shear strength) of two soils from two different lateritic origins and to identify the geomineralogical factors that can influence their physical and geomechanical performances.

## 2. Materials and Experimental Procedures

### 2.1. Characteristics of the Raw Materials

**Figure 1:**
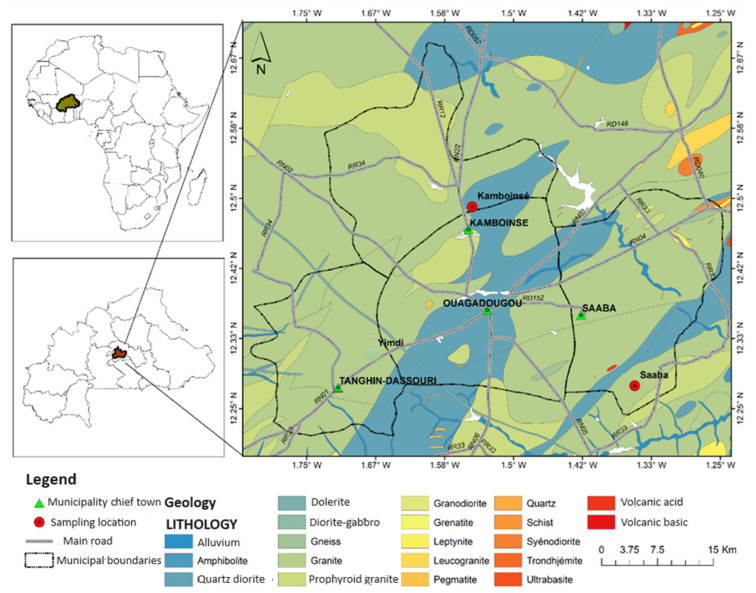
Location of study sites on the geological map of Burkina Faso.

The landscape of the study areas is typical of the central plateau of Burkina Faso, generally flat with Sudano-Sahelian vegetation. The geological map of the region (Figure 1) shows that the bedrock consists of Palaeoproterozoic granitoids from the Leo shield. The surface soils are lightly cemented gravel with sand and fine fractions, characteristic of lateritic soils.

The lateritic soils come from two sites located in the province of Kadiogo (Burkina Faso): Saaba and Kamboinsé. These sites were chosen due to their frequent use in the construction of several road sections (National Roads No. 4 and No. 22, urban roads in the city of Ouagadougou).

**Figure 2:**
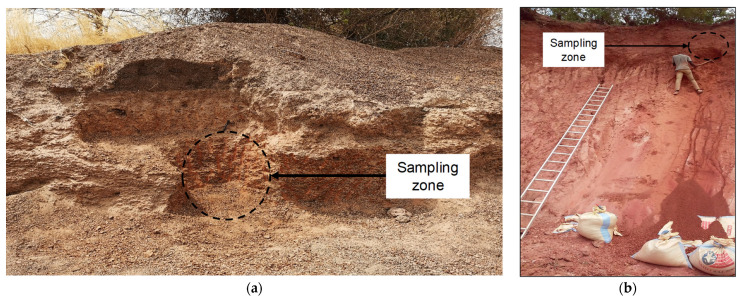
Profiles of lateritic soils: (**a**) Saaba (LAS) and (**b**) Kamboinsé (LAK).

The site of Saaba, commonly known as Badnogo 2 (Figure 2a), is located 21 km east of Ouagadougou (12°16′37.5″ N, 1°21′12.1″ W). The site covers an area of around 0.3 km^2^, and its thickness is approximately 2 m. The site of Kamboinsé, known as Saam-tanga (Figure 2b), is located 15 km north of Ouagadougou (12°29′23.71″ N, 1°32′59.35″ W). The site covers an area of approximately 0.14 km^2^, and its thickness is 13 m.

For these types of soil, the horizon of choice for road engineers is the crusts or duricrusts, which are located on the surface and have very good qualities for road construction [29]. These are ferruginous or calcareous crusts [30]. This horizon is embedded between a layer of fine lateritic soil or gravel of poor quality for road construction and a layer of plant gravel or shell debris [31].

A sampling campaign was carried out on the two lateritic sites. Samples were taken on the laterite front in the mineral horizons, that is, the B horizon, using a pickaxe and shovel (Figure 2). These horizons were identified by observing the texture, colour and structure of the material.

The colours of the constituent materials were described using Munsell’s method [32]. The lateritic soils from the site of Saaba, labelled LAS, were reddish yellow (5YR7/6), while those from Kamboinsé, LAK, were reddish-brown (10R 4/6). Appropriate quantities of the material for laboratory tests were collected and stored in bags and hermetically sealed containers to avoid variations in the natural water content of the materials.

The mineralogical composition of the two soils determined by X-ray diffraction (XRD) is presented in Table 1. These soils are mainly composed of hematite, goethite, kaolinite, quartz, k-feldspars and illite. These minerals are the most encountered in lateritic soils [24,33,34]. These results reveal the absence of swelling minerals such as smectites, which can increase plasticity and act negatively on the mechanical properties of materials. LAS contains more clay minerals (64%) than LAK (58%).

**Figure 3:**
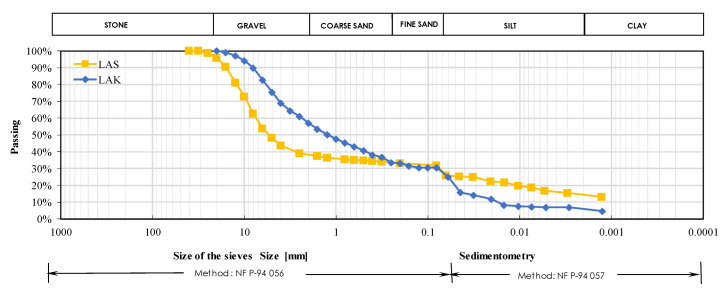
Grain size distribution curve of the two lateritic soils (LAS and LAK).

The grain size distribution curves for these soils are shown in Figure 3. The physical and mechanical properties of these soils are presented in Table 2. These soils have different classes: LAS is clayey gravel (GC), whereas LAK is clayey sand (SC), according to the USCS (Unified Soil Classification System) classification. The increase in fine particles (<80 µm) after the CBR test shows that LAK (8.5%) is crumblier than LAS (2%). The plasticity modulus (m*IP) is used to assess the effective contribution of the plasticity of fines to the performance of road construction materials. LAS has a lower value of plasticity modulus (516) than LAK (726), and therefore its plasticity has a lower influence on its mechanical performance than LAK. The CBR indices of LAS and LAK after 4 days of immersion are 16% and 49%, respectively.

According to CEBTP [6], LAS can be used as a backfill (CBR > 10%) and LAK as a sub-base (CBR > 30%). It therefore seems necessary to improve the physical and mechanical characteristics of these soils so that they can be used as base courses for pavements. Therefore, the improvement with cement is more suitable as these materials have low plasticity and a low clay content. Table 2 summarises more engineering characteristics of the lateritic soils.

The hydraulic binder CEM II/A-L 42.5R, produced by CIMFASO (Ouagadougou, Burkina Faso), was used for the treatment of the soils. The specific gravity of the cement is 3.15. According to the guide to earthworks and road bases [6], this type of cement can be used for soil treatment.

### 2.2. Experimental Procedures

**Figure 4:**
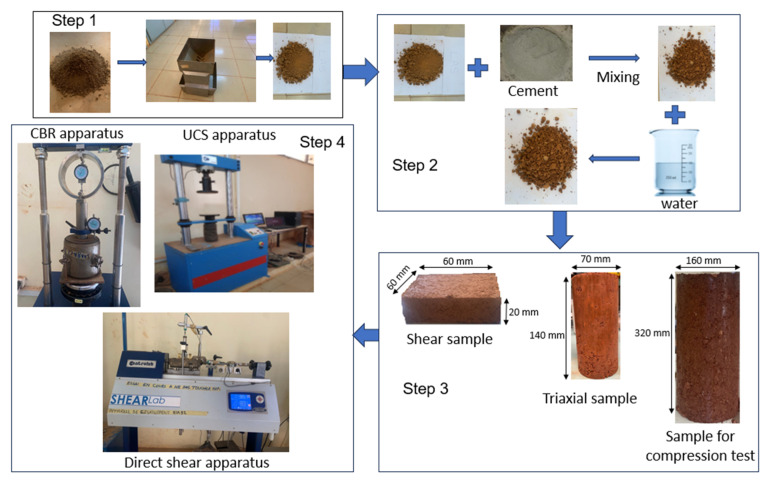
Experimental procedures.

As shown, the experimental procedures followed the steps described in Figure 4. (Step 1) The soil was quartered beforehand to avoid grain segregation. (Step 2) The soil sample obtained after quartering was carefully mixed with cement at different percentages (1%, 2% and 3% of the mass of dry soil) until homogenisation. The mixtures are then thoroughly mixed, adding a quantity of water corresponding to the optimum water content (OWC) of the modified Proctor, and used to make various specimens for the mechanical tests (Step 3 and Step 4).

#### 2.2.1. Physical and Compaction Properties

The Atterberg limit test was carried out in accordance with the standard [35] on the fraction of materials less than 400 µm. The methylene blue value (VBS) was carried out in accordance with the standard [36] on the fraction of materials below 80 µm. The modified Proctor test is used to determine the optimum moisture content and the maximum dry density, in accordance with the standard [37], on fractions finer than 20 mm. These compaction characteristics served as a reference for making the test specimens used to determine the mechanical parameters of the soils studied.

#### 2.2.2. Characterisation of the Mechanical Properties

The CBR test is carried out in accordance with the standard [38]. The general principle of the test is to measure the load to be applied to a cylindrical punch to make it penetrate a sample of material at a constant speed (1.27 mm/min). The test allowed for the determination of 3 different parameters: the immediate CBR index, the CBR index after four days of soaking the sample in water, and the CBR index after three days of curing in air and four days of soaking in water.

The unconfined compressive test was carried out in accordance with the standard [39] on cylindrical specimens measuring 160 mm in diameter (D) and 320 mm in height (H), yielding a slenderness ratio H/D of 2. The specimens were compacted at the compaction energy and optimum water content of the modified Proctor test. Compaction was carried out in 16 layers of 54 blows of 14 kg each. All these specimens were stored for 7 and 28 days in airtight plastic bags at 20 ± 2 °C. The test was used to determine the simple compressive strength ($R_{c}$) of lateritic soils and the Young’s modulus (E_30_) using relationships (1) and (2), respectively, in accordance with standards [39,40]. $R_{c}$ (MPa) is the compressive strength of the specimen, $F_{r}$ (N) is the maximum force supported by the specimen and $A_{c}$ (mm^2^) is the cross-sectional area of the specimen. E (MPa) is the modulus of elasticity in compression, D (mm) is specimen diameter and ε (%) is the longitudinal elongation of the specimen when $F=0.3\times F_{r}$.

(1) $$R_{C}=\frac{F_{r}}{A_{C}}$$

(2) $$E=\frac{\sigma }{\varepsilon }=\frac{1.2\times F_{r}}{\pi D^{2}\varepsilon }$$

As specified in the standard [41], the indirect tensile test consists of applying a lateral compressive load along two opposite generators to a cylindrical specimen until failure. The specimens were moulded at the optimum water content. The specimens were compacted manually using modified Proctor energy. The test specimens were prepared in modified Proctor moulds (152 mm in diameter and 152 mm in height, i.e., a slenderness of 1) and compacted using modified Proctor energy. They were then stored in plastic bags at 20 ± 2 °C. The curing times were 7 and 28 days, as for the compression test. Indirect tensile strength was obtained from relationship (3) in accordance with the standard [41]. $R_{it}$ (MPa) is the indirect tensile strength, F (N) is the maximum breaking force, H (mm) is the specimen length and D (mm) is the specimen diameter. The tensile strength ($R_{t}$) is equal to 80% of the indirect tensile strength. It is therefore obtained from the relationship in Equation (4).

(3) $$R_{it}=\frac{2F}{\pi HD}$$

(4) $$R_{t}=0.8R_{it}$$

The Casagrande box shear test was carried out in accordance with the standard [42]. The test was carried out in a consolidated, drained condition, which corresponds to slow shear. It consisted of applying a constant vertical force to a soil sample placed in two independent half-boxes. Once the sample has been consolidated by the vertical applied force, it is sheared along a horizontal plane known as the shear plane, imposed by the relative sliding of the two half-boxes.

The test specimens for the shear test were made to match the dimensions of the half-boxes of the shearing apparatus. They were 60 mm square and 20 mm thick. The only particle fraction of soil tested was less than 5 mm. The samples were moulded using modified Proctor energy. They were formed by static, double-sided compaction. After preparation, the specimens were stored in plastic bags at 20 ± 2 °C for 7 days. When the soil sample was placed in the two half-boxes, the frame was filled with water to achieve saturation. For each type of design, three tests were carried out at vertical stresses of 50, 100 and 200 kPa. As the tests were carried out under consolidated, drained conditions, the settlement values were recorded. The consolidation was complete when the settlement was constant. Once the settlements were stabilised, the sample was sheared at a speed of 0.025 mm/min (slow shear). The test was stopped when the variation in shear force for a measurement interval corresponding to a horizontal displacement δl of 0.5 mm was less than 1/100 of the maximum force.

From the results of the shear test, Mohr-Coulomb failure envelopes were plotted, and c′ (cohesion) and φ′ (internal angle of friction) were determined. The volume variation of the specimens was also determined in order to assess the expansion and contraction behaviour of the specimens during the tests.

## 3. Results and Discussion

### 3.1. Effect of Cement on the Plasticity and Compaction Parameters of Soils

Table 3 presents the results of the Atterberg limits (plasticity limit, liquidity limit and plasticity index) and the modified Proctor test (optimum water content and dry density). The plasticity index decreases with increasing cement content. For LAS and LAK, the plasticity index varies from 15% to 13% and from 19% to 12%, respectively, when the cement content increases from 0 to 3%. The liquid limit increases from 28% to 44% for LAS and from 47.5% to 55% for LAK with 0 to 3% cement. The same is true for the plasticity limit, which increases from 13% to 31% and from 28.5% to 43% with 0 to 3% cement, respectively, for LAS and LAK. These results are similar to those in the scientific literature [21,22,24,25]. An increase in the plastic limit generally results in a reduction in the plastic properties of the soil. This reaction is due to the alteration of the water film surrounding the clay minerals in the soil. The calcium ion in lime is divalent and serves to bind soil particles together. This increases the plasticity of the soil and leads to a more open, granular texture. However, some authors have reached the opposite results. The authors of [23] reported that the liquidity limit and plasticity index decrease with increasing cement content. They suggested that this reduction in the liquid limit and plasticity index could be the result of the cationic reaction that flocculates the fine soil particles, leading to a reduction in the clay fraction. The increase or decrease in the liquid limit depends on the type of soil. Soils containing mineral clays, such as kaolinite, show an increase in the liquid limit, while those containing montmorillonites show a decrease in the liquid limit [43].

The decrease in plasticity index is more significant on LAK than on LAS. Indeed, after the addition of 3% cement, the plasticity index decreases by around 13% and 37% for LAS and LAK, respectively. LAK has a lower content (6%) of clay particles (below 2 μm) than LAS (14%). Since hydration reactions involving clay particles lead to a reduction in this fraction, their quantity will be even lower in LAK, making it less plastic. The cement addition has decreased the plasticity index of the soils to a value of less than 15%, which is the upper limit for a soil to be used as a pavement base course [6]. LAS and LAK can be used as a base course after adding 2% cement.

The maximum dry density (MDD) increases as the cement content increases (Table 3). For LAS, the MDD is 20.3 kN/m^3^ for the untreated soil, while it is 20.6 kN/m^3^ after the addition of 3% cement. For LAK, the MDD is 20.3 kN/m^3^ before treatment and 21.0 kN/m^3^ after treatment with 3% cement. Similar behaviour has been reported by other authors [23,44]. This trend can be explained by considering cement as a filler that will interlock between voids and fill them. In addition, cement, in the presence of water, tends to lubricate the soil particles, resulting in denser compaction during the compaction process. An opposite trend was observed for the two soils in terms of optimum moisture content. In fact, the optimum water content decreases with increasing cement content for LAK, whereas it increases for LAS. An increase in optimum moisture content indicates that the treated material will be easier to compact, as it will accept higher humidity and will be less sensitive to variations in moisture content. Both of these trends have been noted in the literature. The authors of [24,25] have reported an increase in optimum water content, which they attribute to the affinity of cement for water. The increase in cement content should be accompanied by an increase in water demand. The authors of [23] reported a decrease in the optimum water content for a lateritic soil in Cameroon after treatment with cement. It was assumed that water was used in the hydration reactions until there was not enough remaining to saturate the solid surfaces, and consequently, the relative humidity in the paste decreased. The two soils LAS and LAK have a density greater than 20 kN/m^3^ and can therefore be used as a base layer, according to [6,45].

### 3.2. Effect of Cement on Mechanical Properties

#### 3.2.1. CBR Index

**Figure 5:**
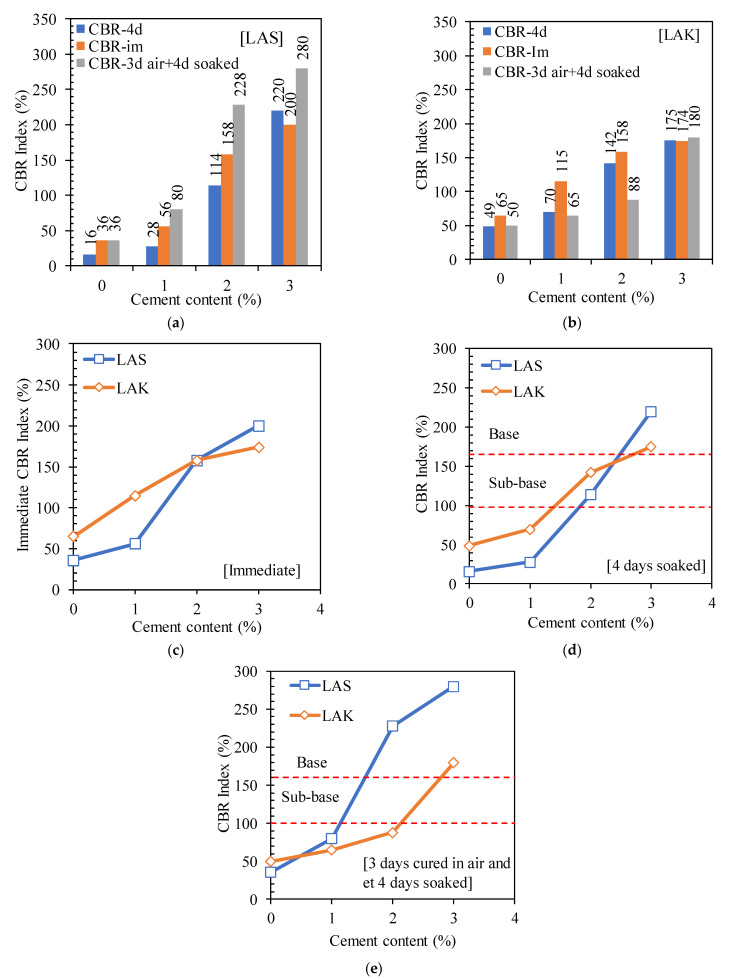
Variation in the CBR index with cement content: (**a**) LAS and (**b**) LAK; (**c**) immediate CBR index; (**d**) CBR index after immersion in water for 4 days and (**e**) CBR index after curing in air for 3 days and immersion in water for 4 days.

The variation in the immediate CBR index (CBR-im), the CBR index after 4 days of immersion in water (CBR-4d) and the CBR index after 3 days of curing in air and 4 days of immersion in water (CBR-3a+4d) of LAS and LAK soils with the addition of cement is shown in Figure 5. Figure 5a,b shows the histograms of variation in these three parameters with cement content for LAS and LAK, respectively. Figure 5c–e shows the variation curves of CBR indices with cement content. In the raw state, the values of CBR-4d for LAS and LAK are 16% and 49%, respectively. The value of CBR-4d of LAK is higher than that of LAS, which could be explained by its lower values of methylene blue (1.33 for LAS and 0.83 g/100 g for LAK) and clay (<2 µm) (14% for LAS and 6% for LAK). The CBR-4d index of LAS is greater than 10%, the minimum value for use in sub-bases. For LAK, it is above 30%, the conventional value for use as a sub-base. However, these two soils cannot be used as base courses.

The CBR indices increase as the cement content increases. For example, the CBR-4d index relatively increased by 1275% and 257% after the addition of 3% cement to LAS and LAK, respectively. This increase in the CBR index reflects the improved bearing capacity of these soils with the addition of cement. This is due to the stiffening of the material by hydration of the cement and the continued development of cementitious compounds such as C-S-H (hydrated calcium silicates) [24]. The author of [27] led to the same conclusions on a Nigerian lateritic soil improved with Portland cement at contents varying from 2 to 10%. The results show that the CBR-im index is higher than the CBR-4d index for all the soils. The CBR-4d index is higher than the CBR-3a+4d index for LAK, while the opposite is observed for LAS. The CBR-4d index, higher than the CBR-3a+4d index for LAK, is due to the uninterrupted hydration process of the cement. After 3 h, the cement had started to harden, and the effect of hydration would only be beneficial long before hardening occurred. Similar results were reported by [23,24]. The author of [46] reported that the CBR-3a+4d index is higher than the CBR-4d (LAS) index. This tendency may be due to the high quantity of clay minerals in the materials studied by Messou. In the present study, LAS contains more clay minerals than LAK, which explains this significant difference.

The CBR-im and CBR-4d indices of LAK were higher than those of LAS for the untreated soil, and the soil improved by 1% and 2% cement, respectively. Since the initial bearing capacity of LAK was higher, the same trend remained even after the improvement with 1% and 2% cement. However, LAS showed higher CBR indices at 3% cement. Given that LAS has a higher content of clay minerals, the amount of 3% cement is high enough to allow additional pozzolanic reactions. This could lead to greater strength in this soil after the addition of 3% cement.

After the addition of 3% cement, LAS and LAK can be used as pavement base courses. Their CBR-4d values with 3% cement are 220% and 175% for LAS and LAK, respectively. These values are higher than 160%, which is the minimum value required for use in base courses, according to [6].

#### 3.2.2. Unconfined Compressive Strength

##### Effect of Cement Content and Curing Time on Unconfined Compressive Strength

**Figure 6:**
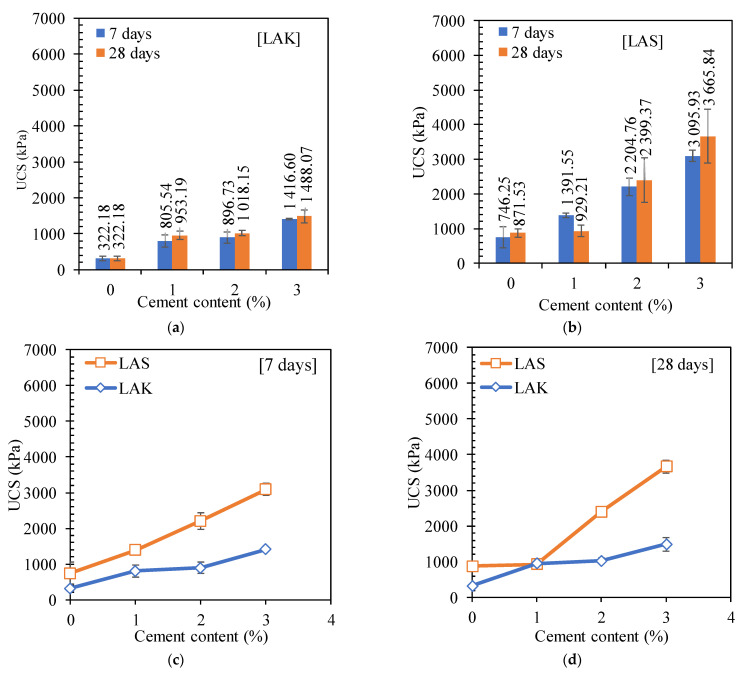
Evolution of the unconfined compressive strength with cement content and curing time: (**a**) LAS and (**b**) LAK; (**c**) comparison between LAS et LAK for a curing time of 7 days and (**d**) comparison between LAS et LAK for a curing time of 28 days.

The evolution of the unconfined compressive strength (UCS) of the two lateritic soils with different cement contents at different curing times is shown in Figure 6. Overall, the UCS increases with increasing cement content.

Figure 6a,b shows that the UCS increases from 746 kPa to 3096 kPa and from 322 kPa to 1488 kPa after the addition of 3% cement for a curing time of 7 days, respectively, for LAS and LAK. It can also be observed that UCS increases with the curing time. However, this increase is much more significant for LAS than for LAK. Indeed, for a cement content of 3%, the relative increase in UCS with curing time is 18% for LAS and 5% for LAK. This increase in strength with the cement content and curing time can be due to several factors. The addition of cement to the soil leads to its hydration and the formation of hydrated calcium silicates, portlandite and other products. Moreover, the pozzolanic reaction involving clay minerals and portlandite leads to the formation of secondary hydrated calcium silicates. These components are responsible for the hardening of the soil–cement mixture.

Figure 6b,c presents the variation curves of the UCS of the two lateritic soils. LAS has the highest UCS compared to LAK. For a cement content of 2%, the UCS of LAS (2399 kPa) is two times higher than that of LAK (1018 kPa). This difference may be due to the fact that LAS already has better physical characteristics than LAK in its raw state. Its plasticity index is lower (15% for LAS compared with 19% for LAK), and its skeleton is more granular. However, the results of the CBR index showed that LAK is harder than LAS (49% compared with 16%). The other explanation may therefore be linked to the higher quantity of mineral clays (kaolinite) in LAS (64% compared with 58%). Mineralogical analysis has shown that LAS contains more mineral clays (kaolinite) than LAK. The hydration of the cement leads to the formation of hydrated calcium silicates (C-S-H) and hydrated calcium aluminates (C-A-H), which are responsible for the cohesion and stiffness of the soil. The clay minerals contained in the studied soils react with portlandite (Ca(OH)_2_) to produce more hydrated silicates and aluminates [47,48]. The higher the amount of kaolinite in the mixture, the higher the reactivity. Similar results were shown in the studies of [47]. Other studies have reported UCS values similar to those presented in this study. The authors of [23] reported strengths in the range of 720 to 1910 kPa after the addition of 3% cement to a fine soil in Cameroon. The authors of [49] studied the effect of adding cement and sand to a lateritic soil in Nigeria. For a cement percentage of 3%, the UCS varies between 1000 kPa and 3000 kPa.

The UCS values obtained in the present study are in accordance with the specification mentioned in [6], which requires a strength of between 1800 kPa and 3100 kPa at 7 days of curing for use as a base course. Therefore, LAS can be used as a base course after the addition of 2% cement. After the addition of 3% of cement in LAK, it can be used as a sub-base layer because its strength becomes 1416 kPa, which is higher than the minimum value proposed by [6] (1200 kPa).

##### Effect of Cement Content on the Failure Mechanism of Soils

**Figure 7:**
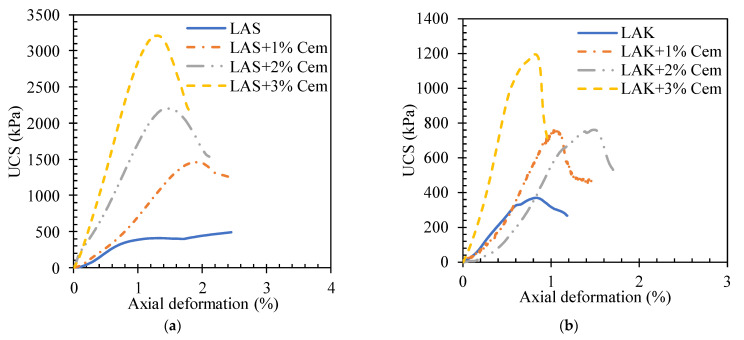
Stress–strain curves at different cement contents for (**a**) LAS and (**b**) LAK.

The stress–strain curves for LAS and LAK before and after treatment with cement for a curing time of 7 days are shown in Figure 7. These results show that the addition of cement influences the mode of failure of the soils. The untreated soil has a ductile strain-hardening mode of failure. It describes a plateau at maximum stress with no remarkable peaks. The curve shows a peak after the addition of 1% cement and beyond. The slope increases as the cement content increases. Failure occurs when the maximum strength is reached. The strength then decreases suddenly and continuously. The behaviour described is similar to that of a brittle material. The axial deformation corresponding to maximum strength is 1.8%, 1.4% and 1.2%, respectively, with cement contents of 1%, 2% and 3% for LAS. For LAK, these strains are 1.1%, 1% and 0.75%, respectively, with cement contents of 1%, 2% and 3%. These strains at maximum stress decrease with increasing cement content. This reflects the increase in soil stiffness with cement content.

#### 3.2.3. Young’s Modulus (E_30_) and Tensile Strength

##### Young’s Modulus (E_30_)

**Figure 8:**
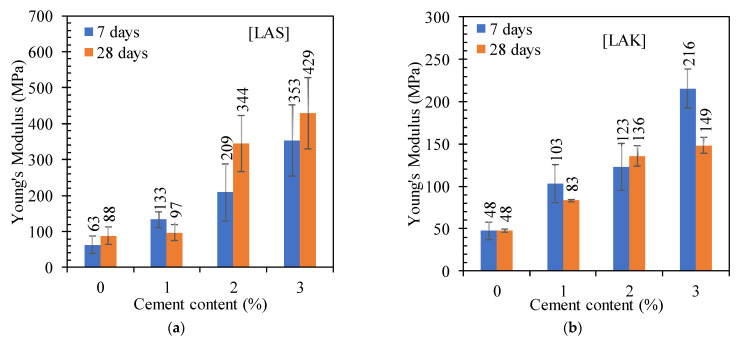

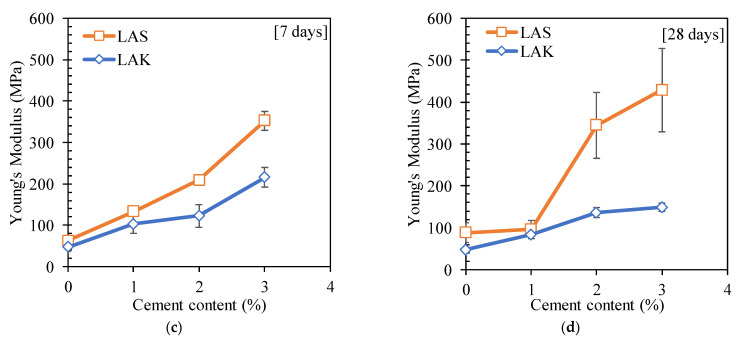
Evolution of the Young’s Modulus with cement content and curing time: (**a**) LAS and (**b**) LAK; (**c**) comparison between LAS et LAK for a curing time of 7 days and (**d**) comparison between LAS et LAK for a curing time of 28 days.

The variation in Young’s modulus is shown in Figure 8. The Young’s modulus increases with increasing cement content and curing time (Figure 8a,b). For LAS, the Young’s modulus increases from 63 MPa to 353 MPa after the addition of 3% cement and 7 days of curing. For LAK, it increases from 48 MPa to 216 MPa after the addition of 3% cement and 7 days of curing. Similarly, the moduli of LAS are higher than those of LAK for all cement contents (Figure 8c,d).

For the same conditions of treatment and curing, the Young’s modulus reported by [23] is lower than that reported in the present study. Indeed, it varies from 54 MPa to 193 MPa and from 68 MPa to 230 MPa for respective curing times of 7 and 28 days. This could be explained by the nature of the soil studied by these authors [23], which is a fine soil containing 34% clay (<2 µm), which is higher than in the present study. In general, cement improvement is less effective when the clay content is high.

According to [20], and as quoted by [33], a lateritic soil with a modulus of elasticity greater than 300 MPa is suitable for use as a road base. After adding 2% cement, LAS has a modulus of 344 MPa after 28 days of curing. LAK does not meet this criterion for all the cement content and curing time.

##### Tensile Strength

**Figure 9:**
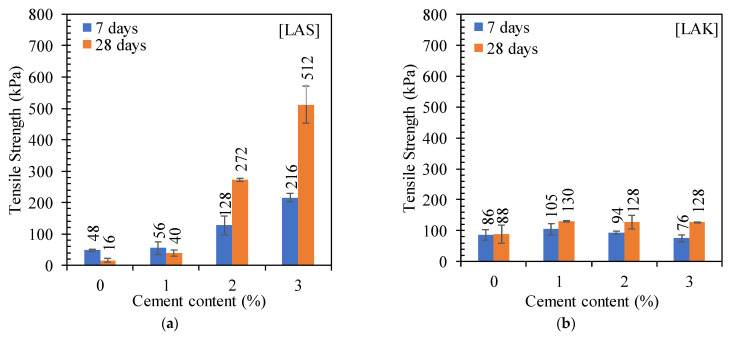
Evolution of the tensile strength with cement content and curing time: (**a**) LAS and (**b**) LAK.

The tensile strength increases with cement content, as shown in Figure 9. The tensile strength increases from 48 kPa to 216 MPa for LAS and from 86 kPa to 105 kPa for LAK, respectively, with 0 to 3% cement. The small variations in LAK’s tensile strength could be explained by its friability. Indeed, when the lateral compression load was applied, lateral splitting of the samples was observed in the interface zones of the compacted layers. The increase in modulus and tensile strength with cement content and curing time is essentially attributed to the formation of C-S-H produced by the cement hydration reaction and the pozzolanic reaction.

These values of tensile strength are in accordance with those reported in the literature. The authors of [24] reported the values of the tensile strength between 50 kPa and 100 kPa with 3% cement after 7 days of curing. The authors of [23] reported tensile strength values less than 300 kPa after treatment with 3% cement. A minimum tensile strength of 300 kPa after 7 days of curing is recommended in [6], but none of the studied soils met this condition.

The modulus of elasticity and tensile strength at 360 days, determined from the empirical relationships (5) and (6) given by [50], are presented in Table 4.

(5) $$\frac{{Rt}_{28\;days}}{{Rt}_{360\;days}}=0.6$$

(6) $$\frac{E_{28\;days}}{E_{360\;days}}=0.65$$

The pairs of E-Rt values obtained at 360 days are placed on the classification chart for materials treated with hydraulic binders [50] to determine their mechanical quality class (SOL Ti).

The standard [50] recommends a minimum class SOL T2 for the use of treated soils in road bases. According to the chart, only LAS soil meets this specification after the addition of 2% cement. LAK, after the addition of 3% cement, is class SOL T1 and can be used as a sub-base for low-traffic roads.

#### 3.2.4. Direct Shear Strength

##### Stress–Strain Curves

**Figure 10:**
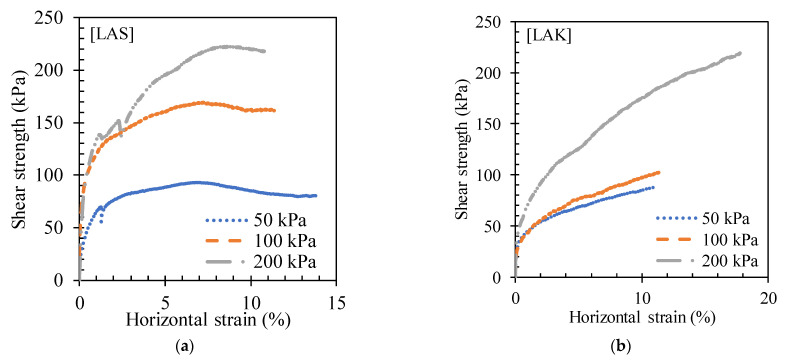
Variation curves of tangential stress as a function of horizontal strain for (**a**) LAS and (**b**) LAK.

**Figure 11:**
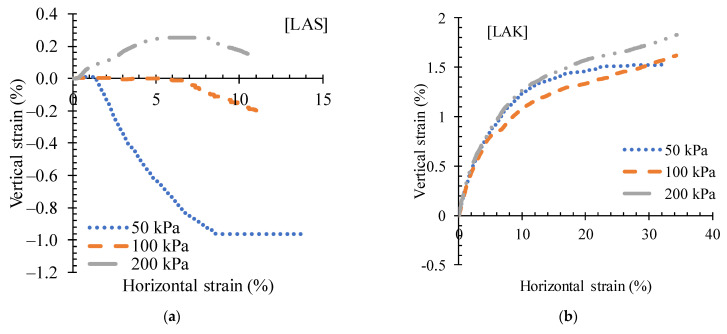
Variation curves of vertical strain versus horizontal strain for (**a**) LAS and (**b**) LAK.

The variation in shear stress with horizontal strain for LAS and LAK in the untreated state is shown in Figure 10. The shear stresses increase with the applied normal stress. At a certain value of shear stress, the curves describe a plateau. The shapes of these curves are characteristic of the behaviour of loose soils [51]. Figure 11 shows the variation in the vertical axial strain with the horizontal strain. LAS changes from an expanding to a contracting state at 1.38% and 2.42% of horizontal strain for normal stresses of 50 kPa and 100 kPa, respectively, whereas it remains expanding for a normal stress of 200 kPa (Figure 11a).

**Figure 12:**
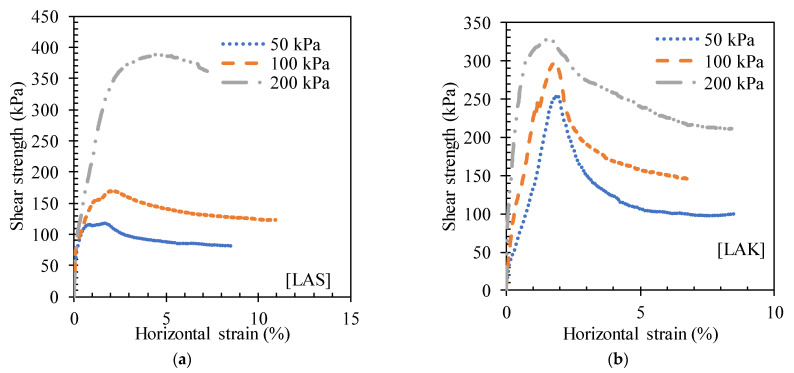
Shear strength versus horizontal strain curves for (**a**) LAS and (**b**) LAK improved with 3% cement.

Figure 11b shows that LAK keeps expanding throughout the test. After the addition of 3% cement to the LAS and LAK lateritic soils, the variation in tangential stress with horizontal deformation is shown in Figure 12. The curves show a peak at maximum stress, illustrating the behaviour of dense and therefore brittle soil. For LAS, the failure occurs at horizontal strains below 5% (2.2% at 50 kPa, 1.6% at 100 kPa and 4.5% at 200 kPa). For LAK, the failure occurs at horizontal strains less than 2% (1.9% at 50 kPa, 1.8% at 100 kPa and 1.6% at 200 kPa).

**Figure 13:**
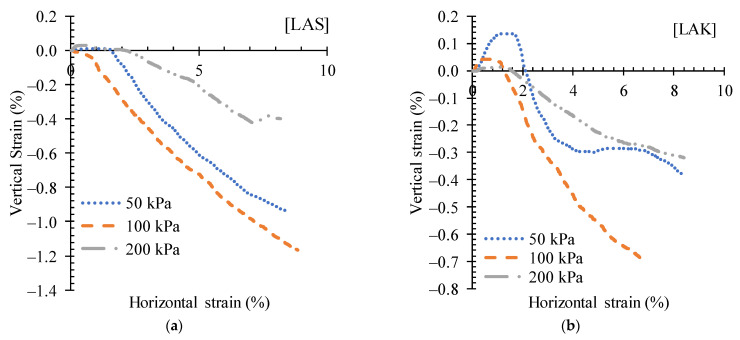
Variation curves of vertical versus horizontal deformation of (**a**) LAS and (**b**) LAK improved with 3% cement.

Figure 13 describes the passage from a contracting state to an expanding state of LAS and LAK soils improved with 3% cement. LAS changes from expanding to contracting at 1.6% and 2.1% of horizontal strain for normal stresses of 50 kPa and 200 kPa, respectively. For a normal stress of 100 kPa, the specimen is contracting throughout the test (Figure 13a). For LAK, the samples change from expanding to contracting at 2.1%, 1.3% and 1.5% for normal stresses of 50 kPa, 100 kPa and 200 kPa, respectively (Figure 13b). The same variations are noted for all cement contents. The maximum shear stresses with normal stresses and the corresponding strains for all cement contents are presented in Table 5. The behaviour described by these materials was noted in the studies of [52] on lateritic soils compacted to optimum water content and saturation.

##### Intrinsic Curves

**Figure 14:**
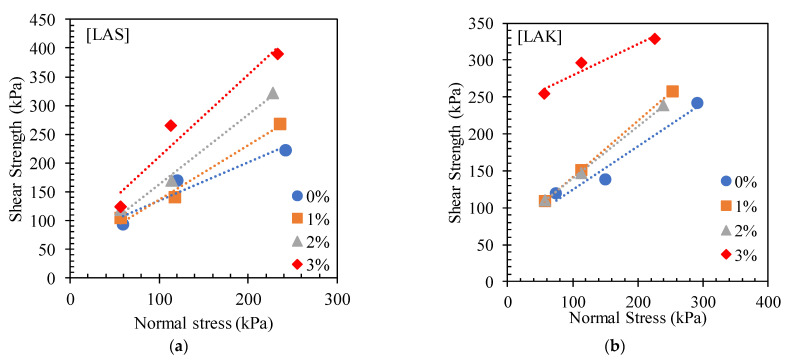
Intrinsic Mohr-Coulomb curves for (**a**) LAS and (**b**) LAK with cement content.

**Figure 15:**
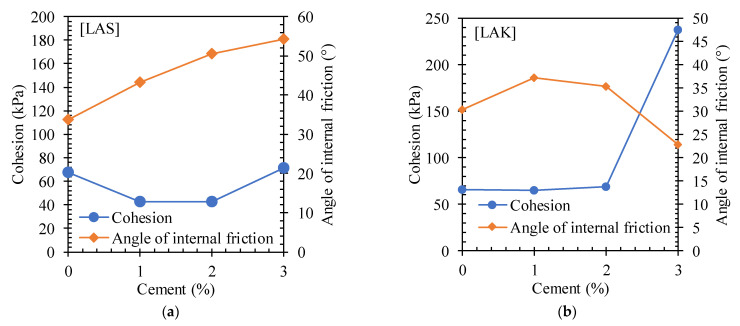
Variation in cohesion and friction angle of (**a**) LAS and (**b**) LAK with cement content.

Figure 14 shows the intrinsic curves for LAS and LAK with cement content. An increase in shear stress was observed with cement content, leading to an upward shift in the intrinsic curves. This shows an increase in the stability zone of the materials and an increase in their load-bearing capacity. Figure 15 shows the variation in the angle of friction of LAS and LAK with cement content obtained from the intrinsic curves. The values of the angles of friction, the cohesion, the maximum shear stress and the corresponding deformations with respect to the cement content of LAS and LAK are presented in Table 5. In the raw state, the friction angles of LAS and LAK are, respectively, 33.7° and 30.3°, while the cohesion values are 67.83 kPa and 65.8 kPa. These values are in the same range as those for Nigerian lateritic materials [53]. The cohesion and angle of friction of LAS are higher than those of LAK. These differences are probably due to the physical parameters of the soils. Granulometric analyses showed that LAS has a higher clay content than LAK (14% compared with 6%), and LAS is more granular than LAK (61.7% and 43%). Consequently, it is more cohesive with a higher angle of friction.

There was an increase in the cohesion of the materials after the addition of 3% cement. For LAS, the cohesion increases from 67.83 kPa to 71.14 kPa with 3% cement. Similarly, the cohesion increases from 65.8 kPa to 237.63 kPa for LAK. However, the angle of friction evolves differently for the two materials. For LAS, the angle of friction increases as the cement content increases. The value is 33.7° before the addition of cement and 54.3° after the addition of 3% cement. For LAK, the angle of friction increases from 30.3° to 37.3° after the addition of 1% cement, and then it decreases to 22.8° after the addition of 3% cement.

The increase in cohesion and friction angle could be explained by the crystallisation phase that follows the hydration of the anhydrous calcium silicates and aluminates in the cement. During this phase, the hydrated constituents coat and bind the grains together, which is known as hydraulic binding. The decrease in the angle of friction of LAK could be due to the internal chemical reactions of certain minerals in the mixture that would not adapt to the presence of cement [54]. Other authors have found the same trend [23,54].

## 4. Conclusions

In this study, the effect of two types of lateritic soils (LAS and LAK) on the effectiveness of geomechanical improvement using a low quantity of cement for sustainable road construction was investigated.

The addition of cement reduces the plasticity index of these soils, increases the maximum dry density and reduces the optimum water content. The CBR indices (immediate, after immersion for 4 days, after 3 days curing in air and after immersion for 4 days) increased. The CBR index after immersion for 4 days increased by 1275% and 257% after the addition of 3% cement for LAS and LAK, respectively. This allowed LAS and LAK to be used as base courses after the addition of 3% cement. LAS has a compressive strength of over 1800 kPa after the addition of cement, and LAK is below this value (1416 kPa), which is the minimum value for use as a base course. Young’s modulus increases by around 460% and 360% for LAS and LAK, respectively, after the addition of 3% cement. Tensile strength also increases with increasing cement content. However, the values are below 300 kPa, which is the minimum value for use as a base course according to CEBTP [6]. The results of the shear tests show that the cohesion and the angle of friction increase with the cement content. However, from the point of view of mechanical behaviour, it appears that the soil changes from a ductile state to a brittle state after the addition of cement. Engineers have to take this change in behaviour into account in their calculation models, despite the low cement content used.

Globally, this study shows that the effect of cement is more significant on LAS soils than on LAK soils. LAS is a clay gravel with low plasticity, while LAK is a clay sand with low plasticity. LAK is more plastic than LAS. LAS contains more fine particles in terms of grain size and a higher quantity of mineral clays. The plasticity modulus m*IP of LAS is less than that of LAK, showing that plasticity has less effect on the mechanical properties of LAS than LAK. Hence, the higher clay content of LAS has no negative effect on its mechanical properties. LAS has better physical properties and a higher mineral clay content. These parameters gave the LAS soil better mechanical properties after treatment. This result is important in that it allows the cement dosage to be optimised based on the mineralogy of the soil, thereby improving the efficiency of the technique.

In the present study, the determination of mechanical properties only concerned static loading, whereas pavement structures are subjected to cyclic loading. To determine the reversible modulus and permanent deformations responsible for rutting in pavement structures, it would therefore be appropriate to study the effect of the addition of cement on these soils using the triaxial cyclic loading test. The durability of soils improved with low doses of cement should be considered.

## Figures and Tables

**Table 1:** Mineralogical composition of the two soils.

Mineral Compositions (%)	LAS	LAK
Quartz	13	17
Goethite	7	14
Hematite	7	7
K-Feldspars	0	4
Tridymite	9	0
Total clay minerals (kaolinite and illite)	64	58

**Table 2:** Engineering properties of the lateritic soils.

Properties	LAS	LAK
Specific gravity of grains (kN/m^3^)	27.8	30.0
Gravel (%)	61.7	43
Sand (%)	13.3	29
Silt (%)	11	22
Clay (%)	14	6
Fine particles (<80 µm)	32	30.5
Increase of fine particles after the CBR test (%)	2	8.5
Dmax (mm)	25	16
ωn (%)	0.73	1.3
Plastic index (%)	15	19
Plastic limit (%)	22	28.5
Liquid limit (%)	37.1	47.5
m*IP = IP*passing of sieve 0.425 mm	516	726
MBV (g/100 g)	1.33	0.83
USCS classification	GC	SC
HRB classification	A2-6	A2-7
OWC (%)	10.9	11.9
MDD (kN/m^3^)	20.3	20.3
Immediate CBR at 95% of MDD (%)	36	65
Soaked CBR at 95% of MDD (%)	16	49

Note: ωn: natural water content, MBV: methylene blue value, OWC: optimum water content, MDD: maximum dry density, CBR: Californian bearing ratio, m*IP: plastic modulus; HRB: American standard Highway Research Board, USCS: Unified Soil Classification System.

**Table 3:** Plasticity and compaction parameters.

Cement Content	Plasticity			Compaction Parameters	
	LL (%)	LP (%)	IP (%)	ɣdopt (kN/m^3^)	ωopt (%)
LAS 0%	28.0	13.0	15.0	20.3	10.9
LAS 1%	34.0	19.0	15.0	20.3	11.4
LAS 2%	42.0	28.0	14.0	20.4	11.4
LAS 3%	44.0	31.0	13.0	20.6	11.4
LAK 0%	47.5	28.5	19.0	20.3	11.9
LAK 1%	56.0	41.0	15.0	20.6	10.8
LAK 2%	56.0	43.0	13.0	20.7	9.1
LAK 3%	55.0	43.0	12.0	21.0	10.3

**Table 4:** Modulus of elasticity, compressive strength and tensile strength of different lateritic soils with cement content and curing time.

Curing Time (Days)		LAS				LAK			
		Cement Content (%)							
		0	1	2	3	0	1	2	3
UCS (kPa)	7	746	1391	2205	3096	322	805	897	1417
	28	871	929	2399	3666	322	953	1018	1488
E (MPa)	7	63	133	209	353	47	103	123	216
	28	88	97	344	428	47	83	136	149
	360	136	149	530	659	73	128	209	228
Rt (kPa)	7	48	56	128	216	86	105	94	76
	28	16	40	272	512	88	130	128	128
	360	27	67	453	853	147	217	213	213

**Table 5:** Shear parameters of LAS and LAK before and after improvement.

		LAS + Cement (%)				LAK + Cement (%)			
σ_n_ (kPa)	Parameters	0	1	2	3	0	1	2	3
50	τ (kPa)	92.98	104.35	118.00	123.86	119.43	108.87	110.70	253.98
100		169.23	139.67	169.74	265.13	138.72	151.44	147.12	296.41
200		222.14	268.04	322.26	389.02	242.23	257.84	238.88	328.86
50	ε_pic (%)_	6.64	1.88	1.65	2.20	25.46	3.61	3.02	1.89
100		7.09	4.98	1.98	1.57	26.37	2.16	2.22	1.77
200		8.22	5.60	2.43	4.48	23.94	12.29	7.26	1.60
	c (kPa)	67.8	42.7	42.6	71.1	65.8	65.0	68.8	237.6
	ϕ (°)	33.7	43.2	50.5	54.3	30.3	37.3	35.3	22.8

## Data Availability

Data will be made available on request.
